# Supplementary material for: CAR affinity modulates the sensitivity of CAR-T cells to PD-1/PD-L1-mediated inhibition
Source: Nat Commun. 2024 Apr 26;15:3552. doi: 10.1038/s41467-024-47799-z (PMC11053011; doi:10.1038/s41467-024-47799-z)
Supplement: Supplementary file 1 — Supplementary Information [file 41467_2024_47799_MOESM1_ESM.pdf]

Supplementary Figure 1

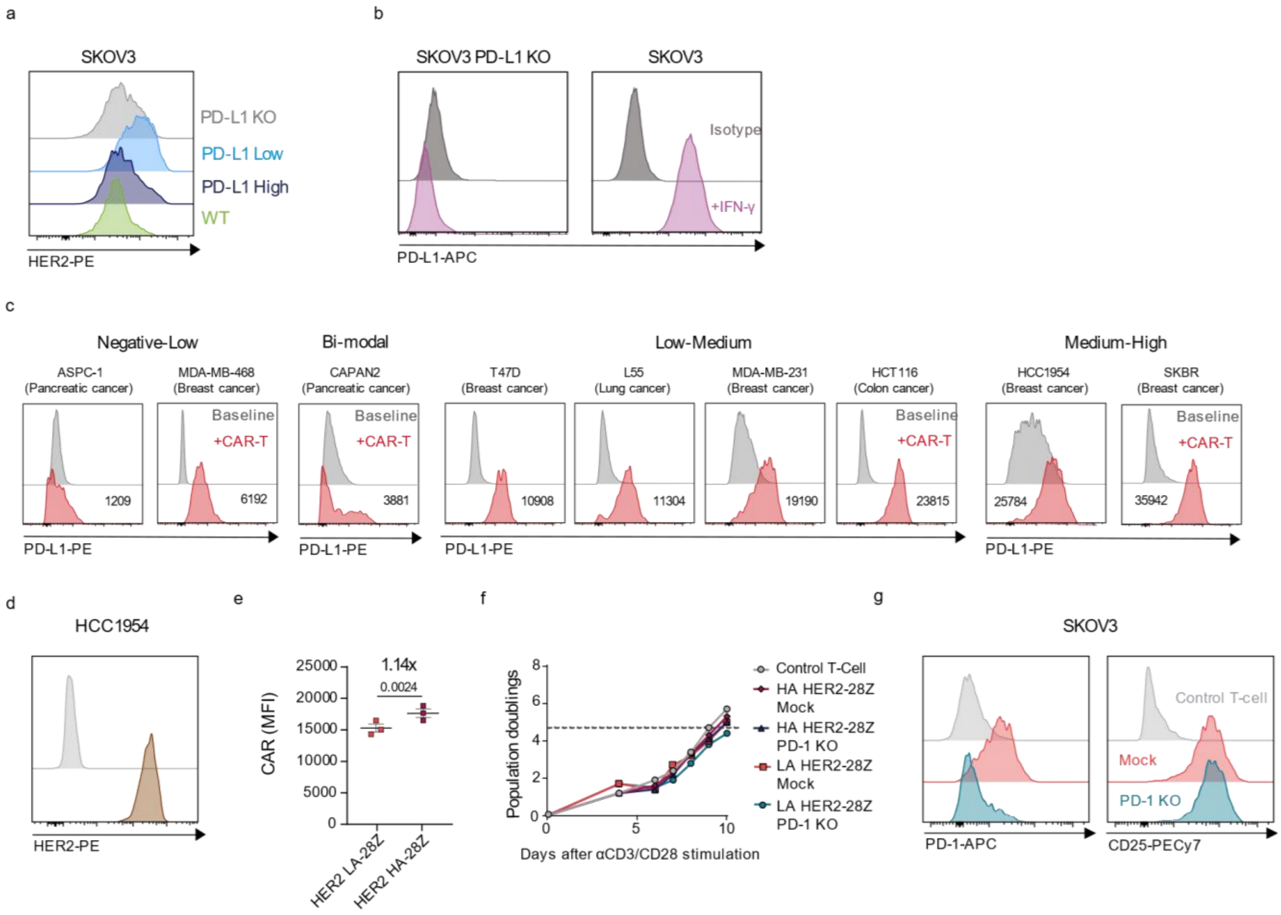

Characterization of the SKOV3 tumor model and assessment of CAR-T cell activation. **a** HER2 expression in SKOV3 cells expressing variable PD-L1 densities as assessed by flow cytometry. **b** Expression and quantification of PD-L1 molecules of a panel of tumor cell lines alone (baseline, gray) or co-cultured with CAR-T cells (red) for 48 hours assessed by flow cytometry. **c** PD-L1 expression by flow cytometry of SKOV3 PD-L1 KO and SKOV3 tumor cells treated with IFN-γ for 48h. **d** HER2 expression in HCC1954 cells using flow cytometry (unstained, gray). **e** Quantification of LA and HA HER-28Z CAR expression by mean fluorescent intensity (MFI) as assessed by flow cytometry (n=3 donors). p value by a two-tailed paired T-test is indicated. **f** Representative population doublings of control T-cells or mock and PD-1 KO LA and HA HER2-28Z measured using trypan blue exclusion during T cell expansion (n=5 donors). **g** PD-1 and CD25 expression gated on LA HER2-28Z and PD-1 KO CAR^{+} T cells by flow cytometry at day 6 after co-culture with SKOV3 wild type cells. Source data are provided as a Source Data file.

Supplementary Figure 2

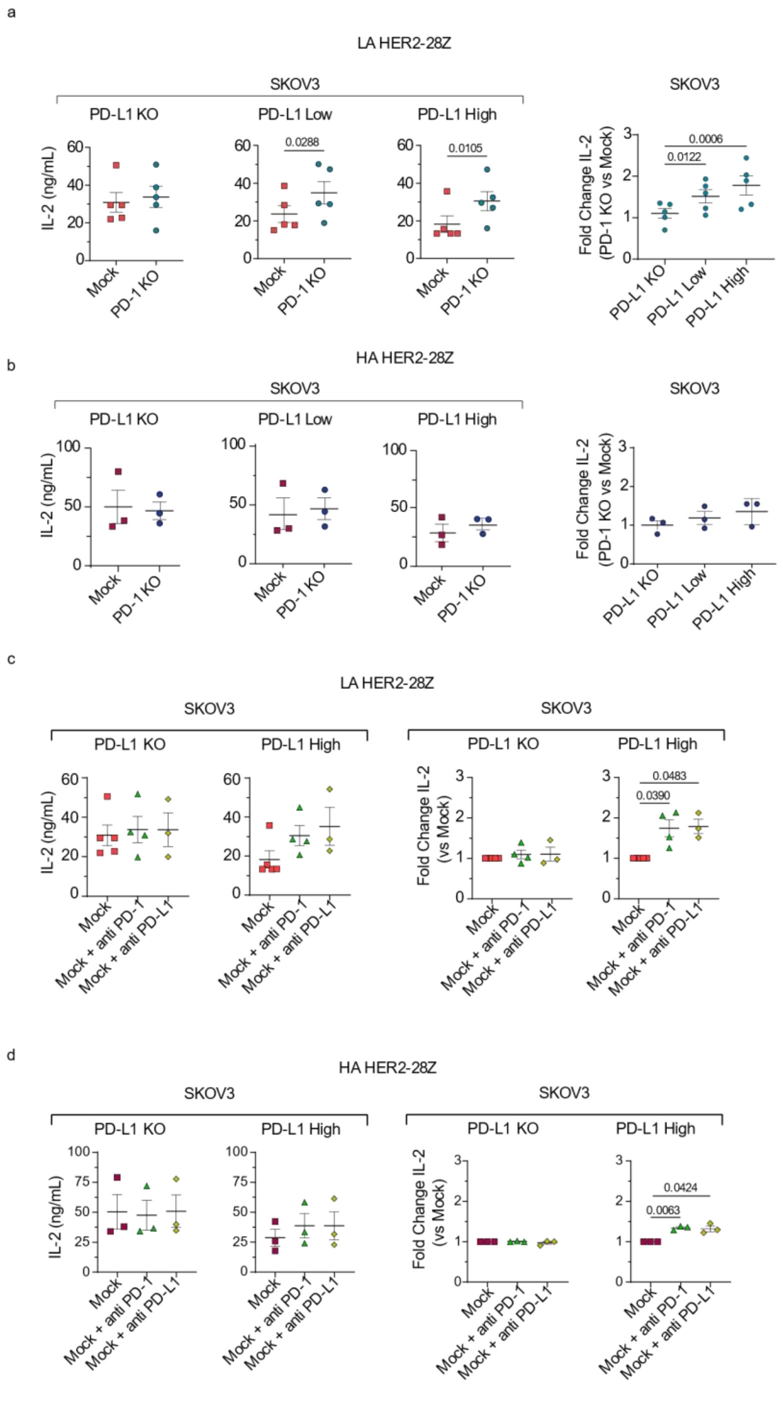

PD-1 KO restores HER2 LA-28Z CAR-T cell IL-2 secretion *in vitro* but does not affect HER2 HA-28Z CAR-Ts. HER2-28Z mock and PD-1-KO CAR-T cells of low **a** or high **b** affinity were co-cultured with SKOV3 tumor cells expressing variable PD-L1 densities (E:T=3:1). IL-2 release was analyzed 24 hours later by ELISA. IL-2 secretion is represented by absolute levels (left panel) or by fold change of PD-1 KO CAR-T cells as compared to mock CAR-T cells (right panel). Data are plotted as mean ± SEM (n=5 donors for LA and 3 donors for HA). p values by two-tailed paired T-test (for absolute levels) or by one-way ANOVA with Tukey post hoc test (for fold change) are indicated. HER2-28Z mock, mock with anti PD-1 or anti PD-L1 CAR-T cells of **c** low or **d** high affinity were co-cultured with SKOV3 tumor cells expressing variable PD-L1 densities (E:T=3:1). IL-2 release was analyzed 24 hours later by ELISA. Cytokine secretion is represented by absolute levels (left panel) or by fold change of mock CAR-T cells with anti PD-1 or anti PD-L1 as compared to mock CAR-T cells (right panel). Data are plotted as mean ± SEM (n=5 donors for mock and 3 donors for mock with anti PD-1 or anti PD-L1 of LA and 3 donors for HA). p values by two-tailed one sample T-test are indicated. Source data are provided as a Source Data file.

Supplementary Figure 3

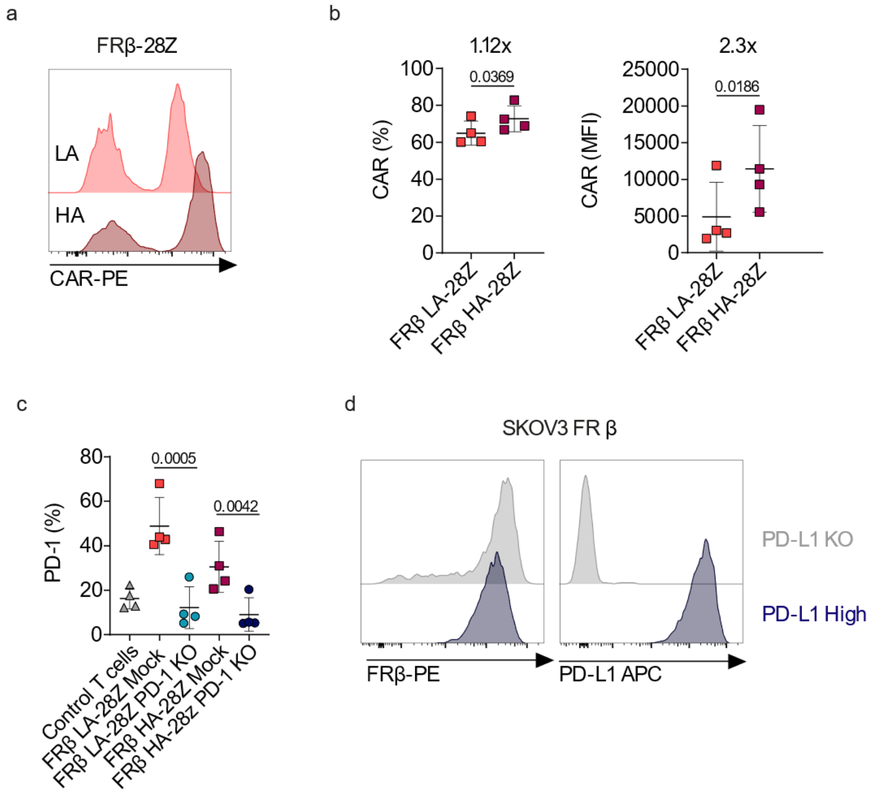

Model based on FRβ-targeting CAR-T cells for evaluation of PD-L1-resistance in a different pair of low/high affinity CARs. **a** Schematic representation of CAR constructs targeting FRβ with either low or high affinity. Flow cytometric analysis showing **b** CAR or **c** PD-1 surface expression of indicated CAR-T cells at day 8 of T-cell expansion. Data plotted as mean ± SD (n=4 donors). P values by two-tailed paired T-test are indicated. **d** FRβ (left panel) and PD-L1 (right panel) expression by flow cytometry in indicated SKOV3 cell lines. Source data are provided as a Source Data file.

Supplementary Figure 4

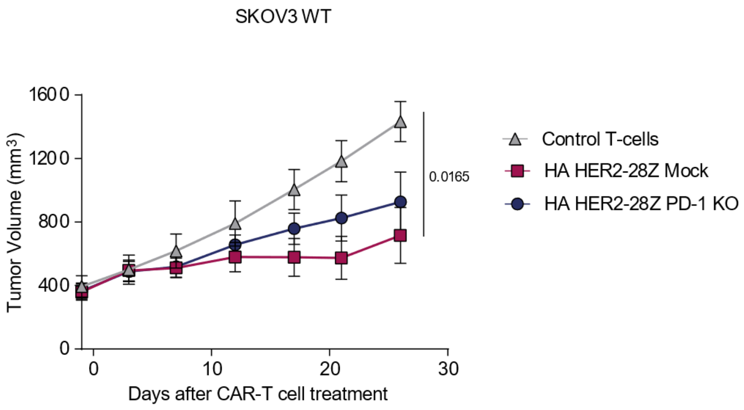

PD-1 KO does not have an impact on HA HER2-28Z CAR-T cells *in vivo*. NSG mice bearing SKOV3 WT were treated with $3x10^6$ control T-cells, mock or PD-1 KO HA HER2-28Z CAR^+-T cells. The mean tumor volume ± SEM is shown (n=6 tumors for control and n=8 tumors for mock and PD-1 KO groups). p value by two-way ANOVA with Tukey's multiple testing correction is indicated. Source data are provided as a Source Data file.

Supplementary Figure 5

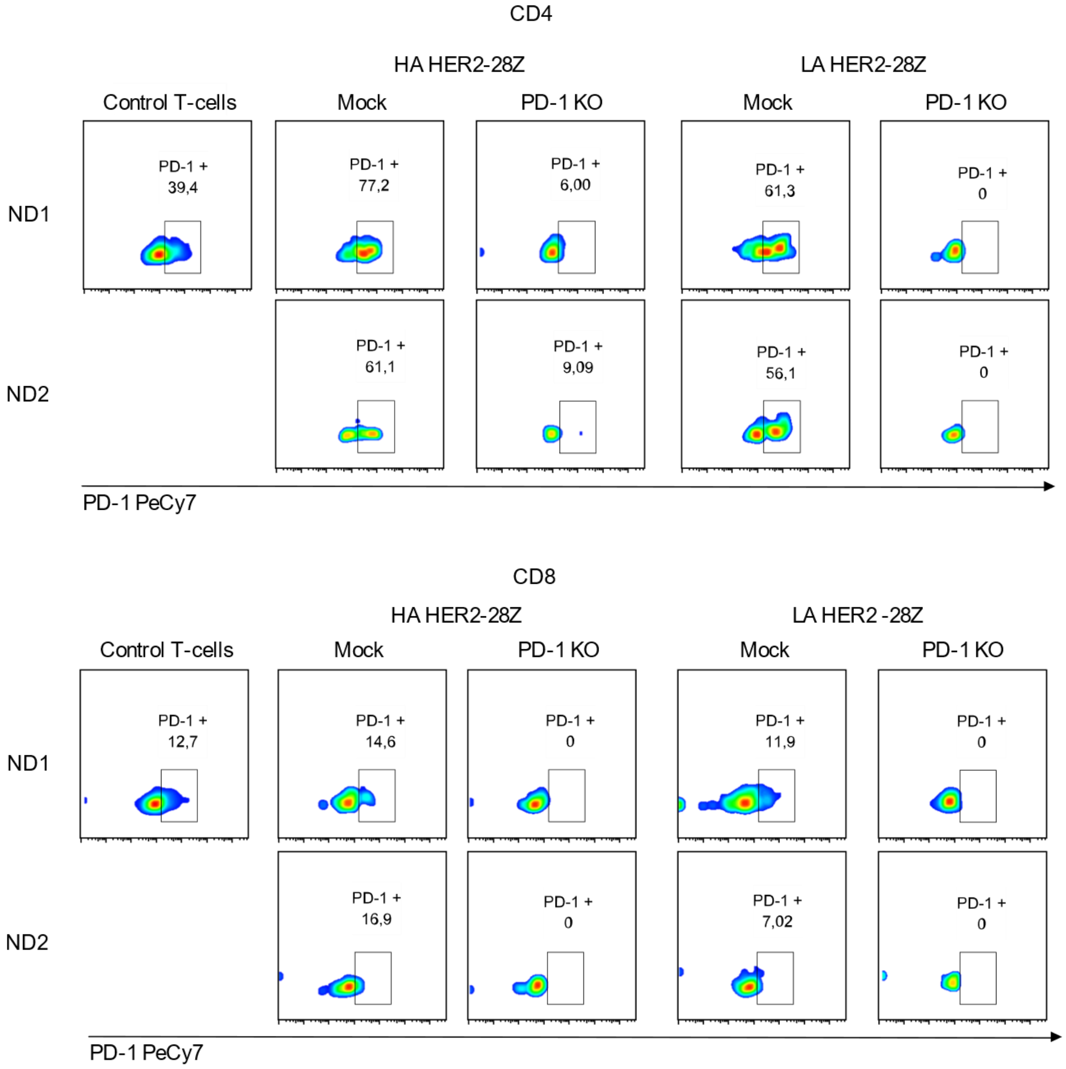

PD-1 expression in LA and HA HER2-28Z CAR-T cells prior to exposure to the bilayers. Surface expression of PD-1 was assessed using flow cytometry on CD4 and CD8 LA and HA HER2-28Z mock and PD-1 KO CAR$^+$ T cells, 16 hours after thawing and just before their introduction into the bilayers. Data for two different donors (ND1 and ND2) is shown. Source data are provided as a Source Data file.

Supplementary Figure 6

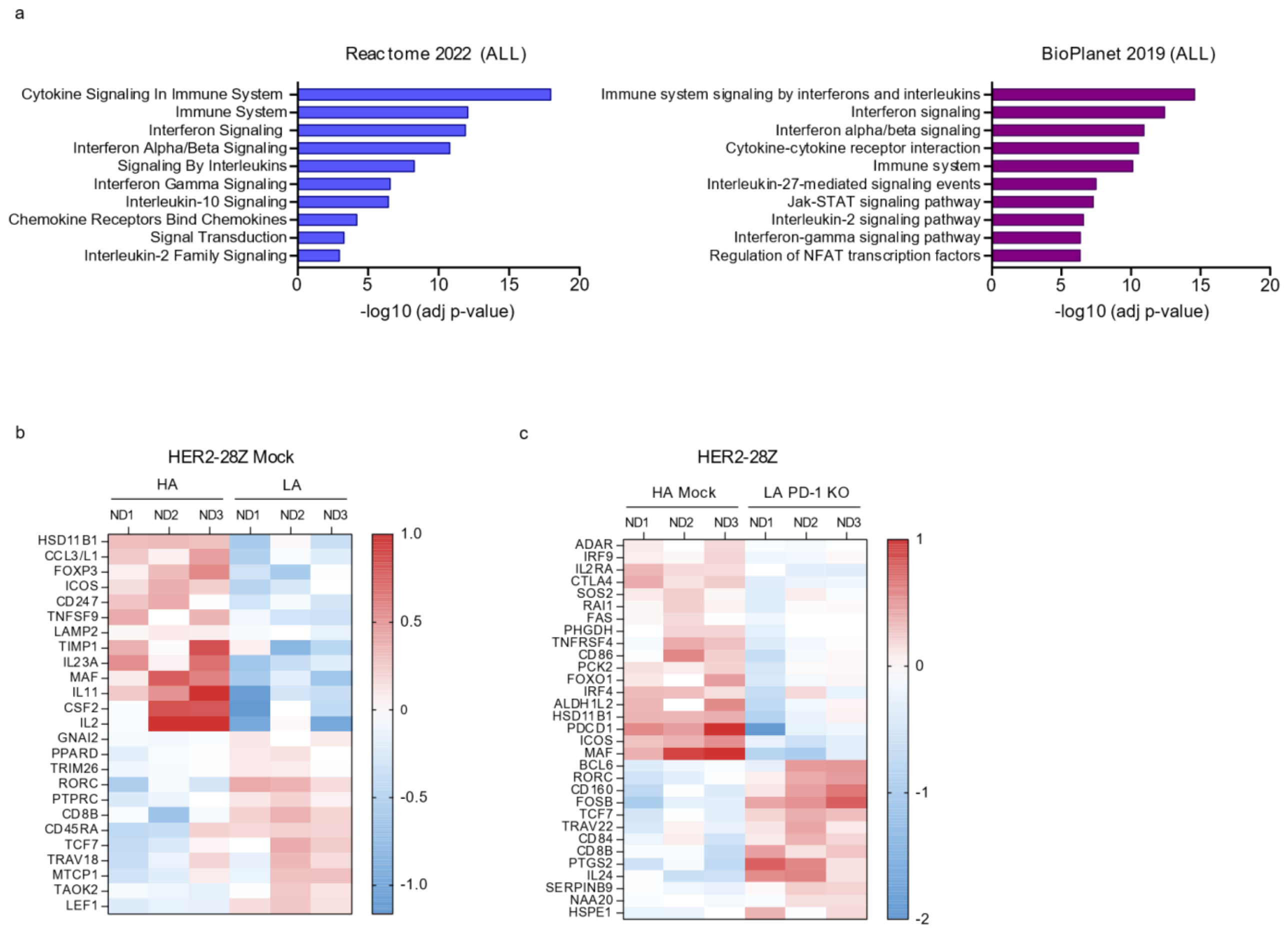

Differential transcriptomic analysis of HER2-28Z CAR-T cells in reference to scFv affinity and PD-1 KO. a Reactome (left panel) and Bioplanet (right panel) pathway analysis of all significant genes between PD-1 KO LA HER2-28Z and mock LA HER2-28Z showing the top-10 enriched pathways. -log10 (adj p-value) was derived from Enrichr. Heat map of statistically significant ($P \leqslant 0.05$) genes that define distinct transcriptional profiles between **b** HA HER2-28Z and LA HER2-28Z CAR T cells and **c** HA HER2-28Z and LA HER2-28Z PD-1 KO CAR T cells after stimulation with SKOV3 tumor cells for 48 hours (n=3 donors). Source data are provided as a Source Data file.

Supplementary Figure 7

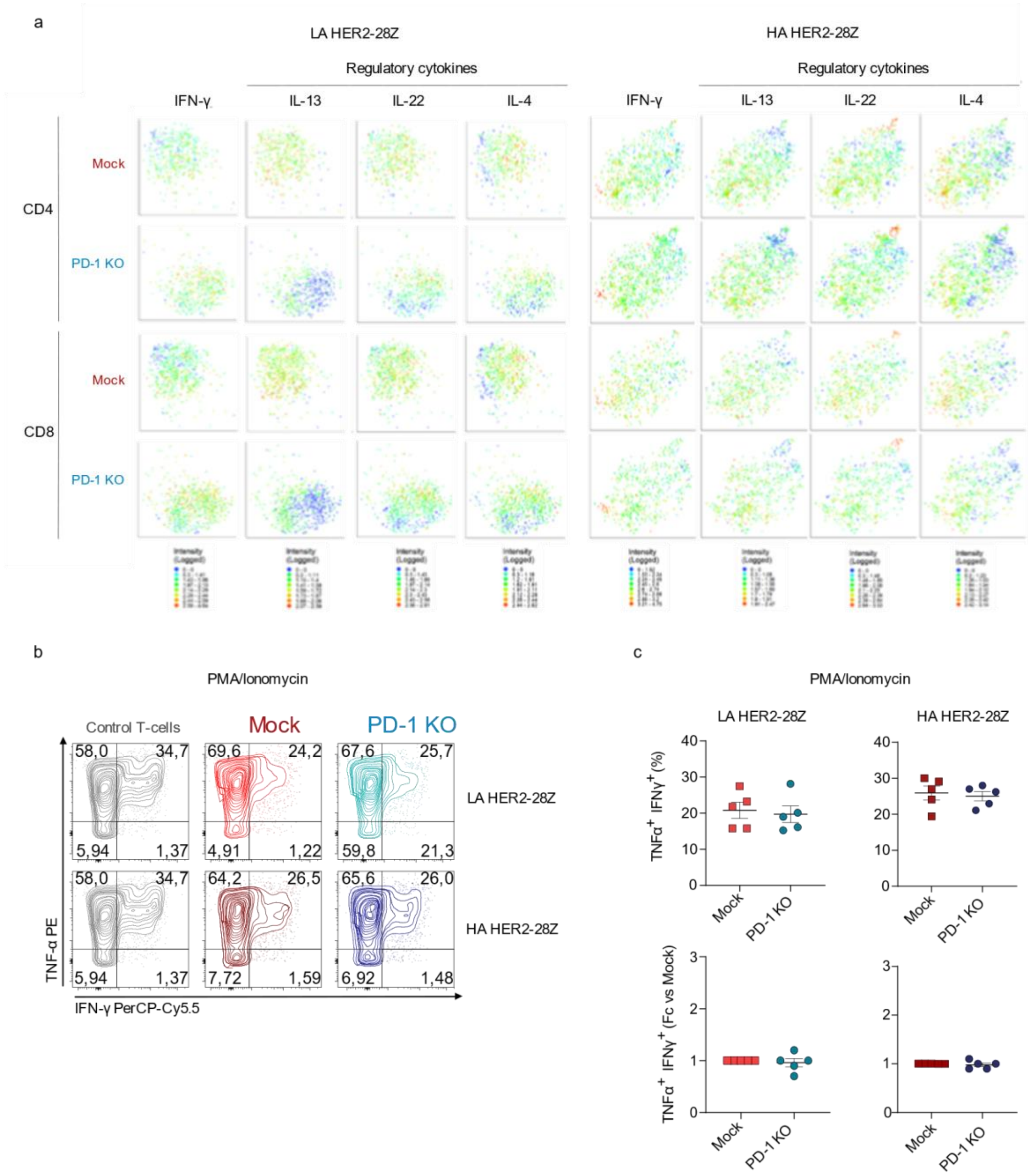

PD-1 KO induce changes in cytokine production profile in LA but not HA HER2-28Z CAR-T cells. Single-Cell Adaptive Immune panel (Isoplexis) of LA and HA HER2-28Z PD-1 KO versus mock CAR-T cells after co-culture with SKOV3 tumor cells (E:T=1:3). **a** Single cell t-SNE plots of IFN-γ and regulatory cytokines (IL-13, IL-22 and IL-4) of LA HER2-28Z mock and PD-1 KO (right panel) or HA HER2-28Z mock and PD-1 KO (left panel). Density scale bar represents marker expression of cytokines for a given cell, ranging from low expression (blue) to high expression (red). **b** Flow cytometry plots of intracellular cytokine staining for TNF-α and IFN-γ in indicated groups after stimulation with PMA-ionomycin (Cell Stimulation Cocktail) for 4 hours. **c** Frequency of IFN-γ^{+}TNF-α^{+} T-cells gated on live/CD45^{+} plotted as mean ± SEM (n=5 donors) are shown. Source data are provided as a Source Data file.

Supplementary Figure 8

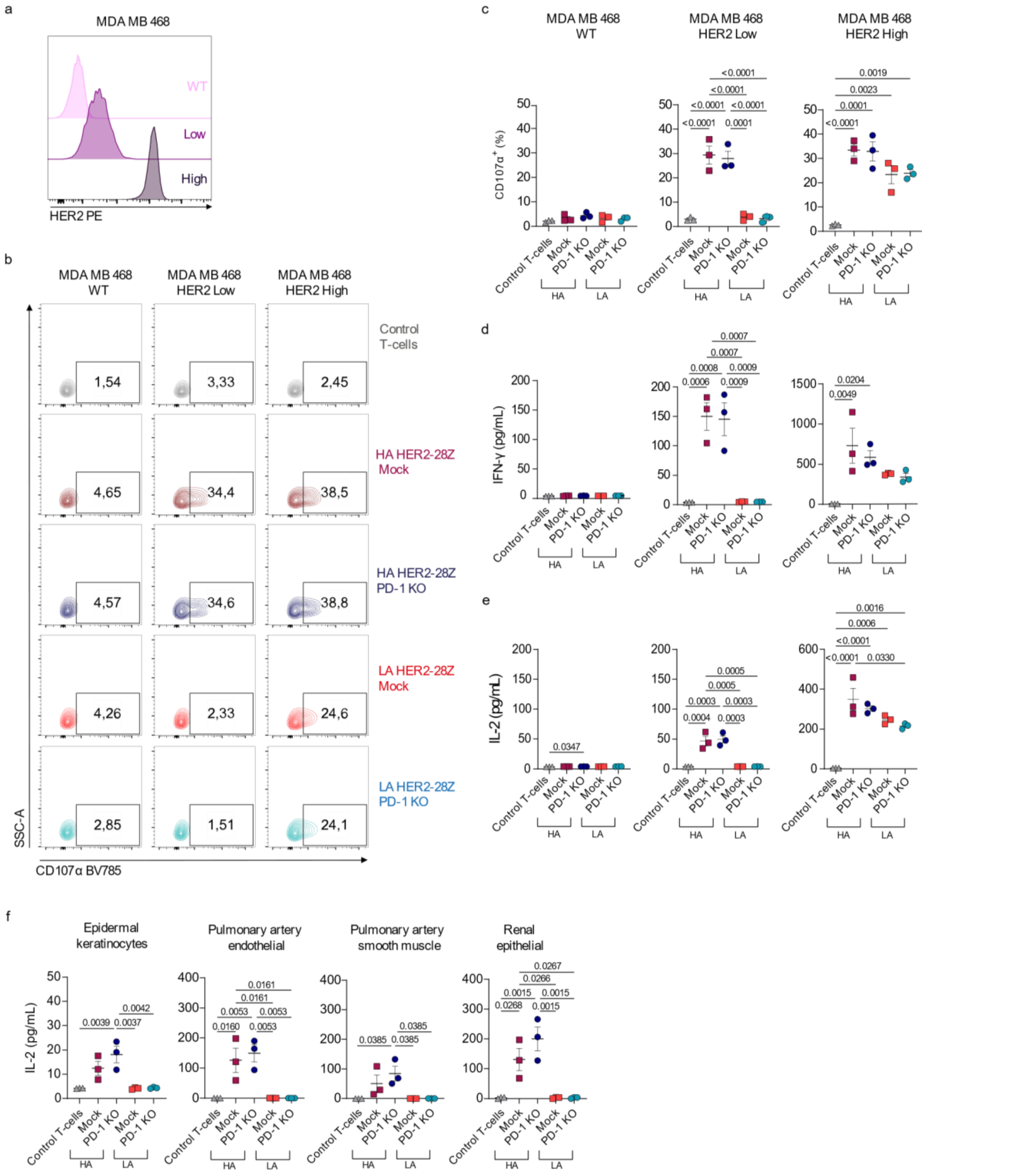

Toxicity profile of mock and PD-1 KO LA and HA HER2-28Z CAR-T cells against a panel of tumor cells expressing variable HER2 densities and of primary healthy cells. a HER2 expression by flow cytometry in wild type MDA-MB-468 cells or MDA-MB-468 cells engineered to express low or high levels of HER2. Mock or PD-1 KO HER2-28Z CAR-T cells of LA or HA were co-cultured with indicated MDA-MB-468 cell lines. CD107-α degranulation marker was measured after 6 hours of co-culture (E:T=1:1). **b** Representative flow cytometry plots and **c** percentage of cells producing CD107-α (gated as live/$CD45^{+}$) plotted as mean ± SEM (n=3 donors) are shown. **d** IFN-γ or **e** IL-2 production by HER2-28Z CAR-T cells after 24 hours of co-culture (E:T=3:1) as quantified by ELISA. **f** IL-2 production by HER2-28Z CAR-T cells after 24 hours of co-culture with a panel of human primary healthy cells (E:T=3:1) as quantified by ELISA. Absolute levels are plotted as mean ± SEM (n=3 donors). p values by one-way ANOVA with Tukey's multiple testing correction are indicated. Source data are provided as a Source Data file.

Supplementary Figure 9

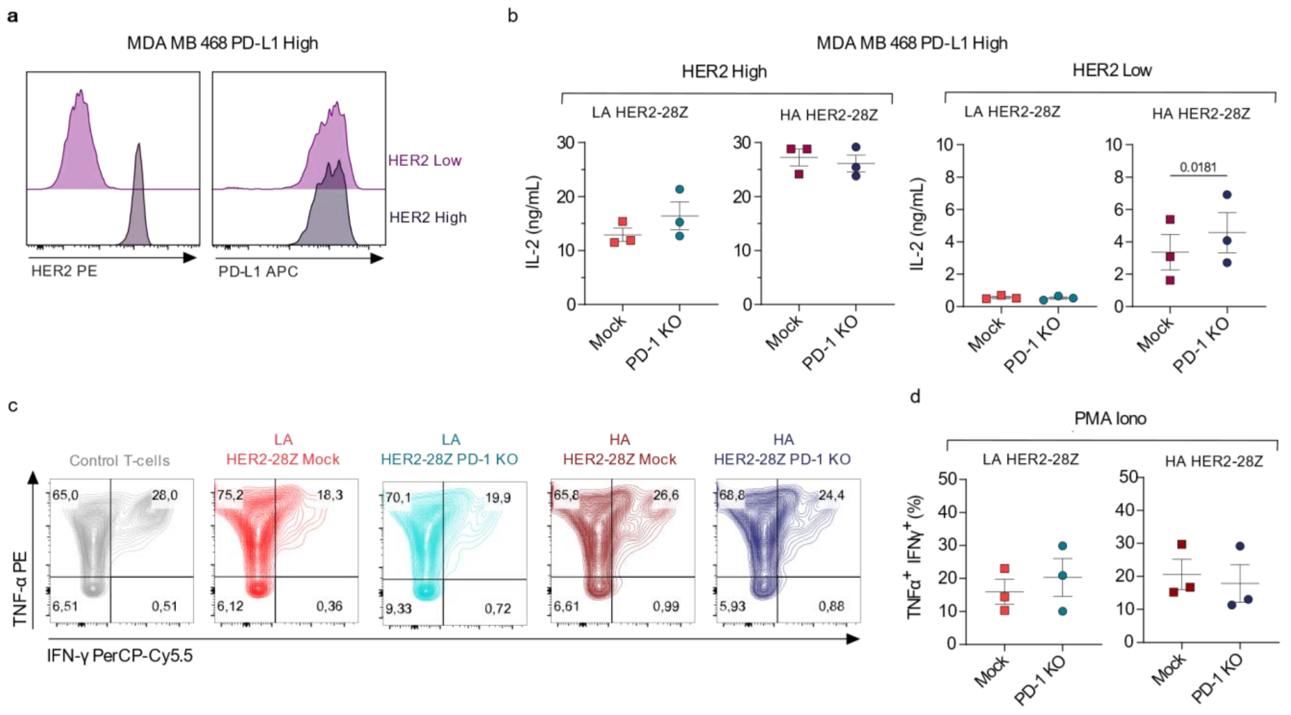

Role of target antigen densities and CAR frequencies in determining sensitivity to PD-L1. a HER2 (left panel) and PD-L1 (right panel) expression by flow cytometry in MDA-MB-468 cells engineered to overexpress PD-L1 and either low or high levels of HER2. **b** IL-2 production by mock or PD-1-KO LA or HA HER2-28Z CAR-T cells after 24 hours of co-culture with MDA-MB-468 PD-L1 high HER2 high (left panel) or MDA-MB-468 PD-L1 high HER2 low (right panel) (E:T=3:1) as measured by ELISA. Mean ± SEM is plotted (n=3 donors). p value by two-tailed paired T-test is indicated. Intracellular cytokine staining for TNF-α and IFN-γ in mock and PD-1 KO LA and HA HER2-28Z CAR-T cells after stimulation with PMA-ionomycin (Cell Stimulation Cocktail) for 4 hours. **c** Representative flow cytometry plots and **d** frequency of IFN-γ+TNF-α+ gated on live/CD45+ T-cells plotted as mean ± SEM (n=3 donors) are shown.

Supplementary Figure 10

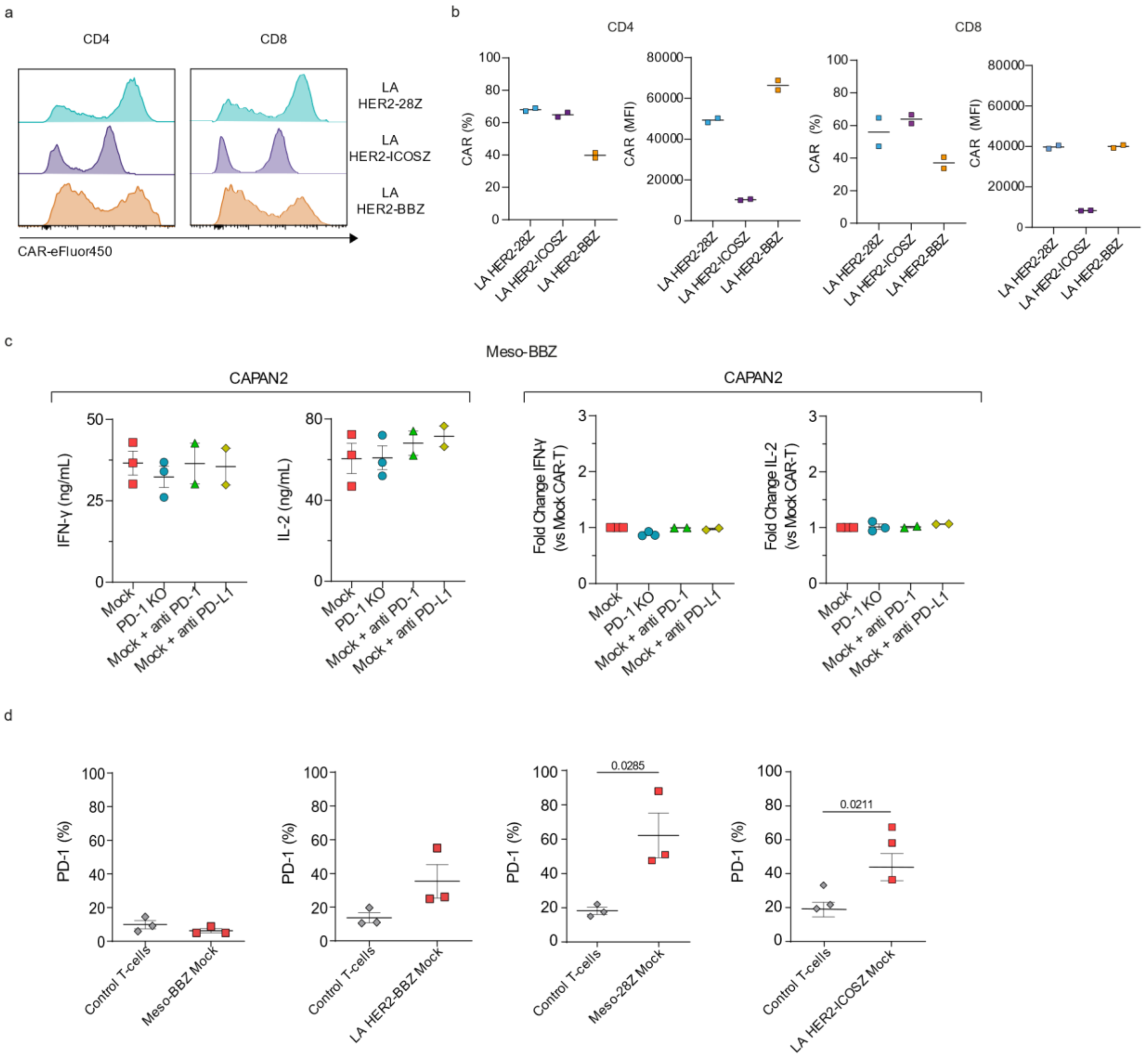

4-1BB costimulatory domain renders M11 CAR-T cells insensitive to PD-1/PD-L1 inhibition. a Representative flow cytometry plots depicting levels of CAR expression by LA HER2 CAR-T cells containing CD28, ICOS or 4-1BB as the co-stimulatory domain. CD4+ T cells (left panel) and CD8+ T cells (right panel) are shown. **b** Quantification of percentage of CAR positive cells and MFI of indicated CARs in $CD4^+$ or $CD8^+$ T cells. Data is represented as mean ± SEM (n=2 donors). p values by one way ANOVA with Tukey post hoc test are shown. **c** M11-BBZ mock CAR-T cells alone or in combination with anti PD-1 or anti PD-L1 antibodies, or PD-1 KO M11-BBZ CAR-T cells were co-cultured with CAPAN2 tumor cells (E:T=3:1). IFN-γ and IL-2 release was analyzed 24 hours later by ELISA. Cytokine secretion is represented by absolute levels or by fold change of indicated groups as compared to mock CAR-T cells. Data are plotted as mean ± SEM (n=3 donors for mock and PD-1 KO and n=2 for mock in combination with antibodies). **d** Percentage of PD-1 expression by flow cytometry of indicated CAR-T cells and their corresponding control T cells on day 8 of T-cell expansion (n=3 donors for all groups except for LA HER2-ICOSZ with n=5). p values by two-tailed paired T-test are indicated. Source data are provided as a Source Data file.

Supplementary Figure 11

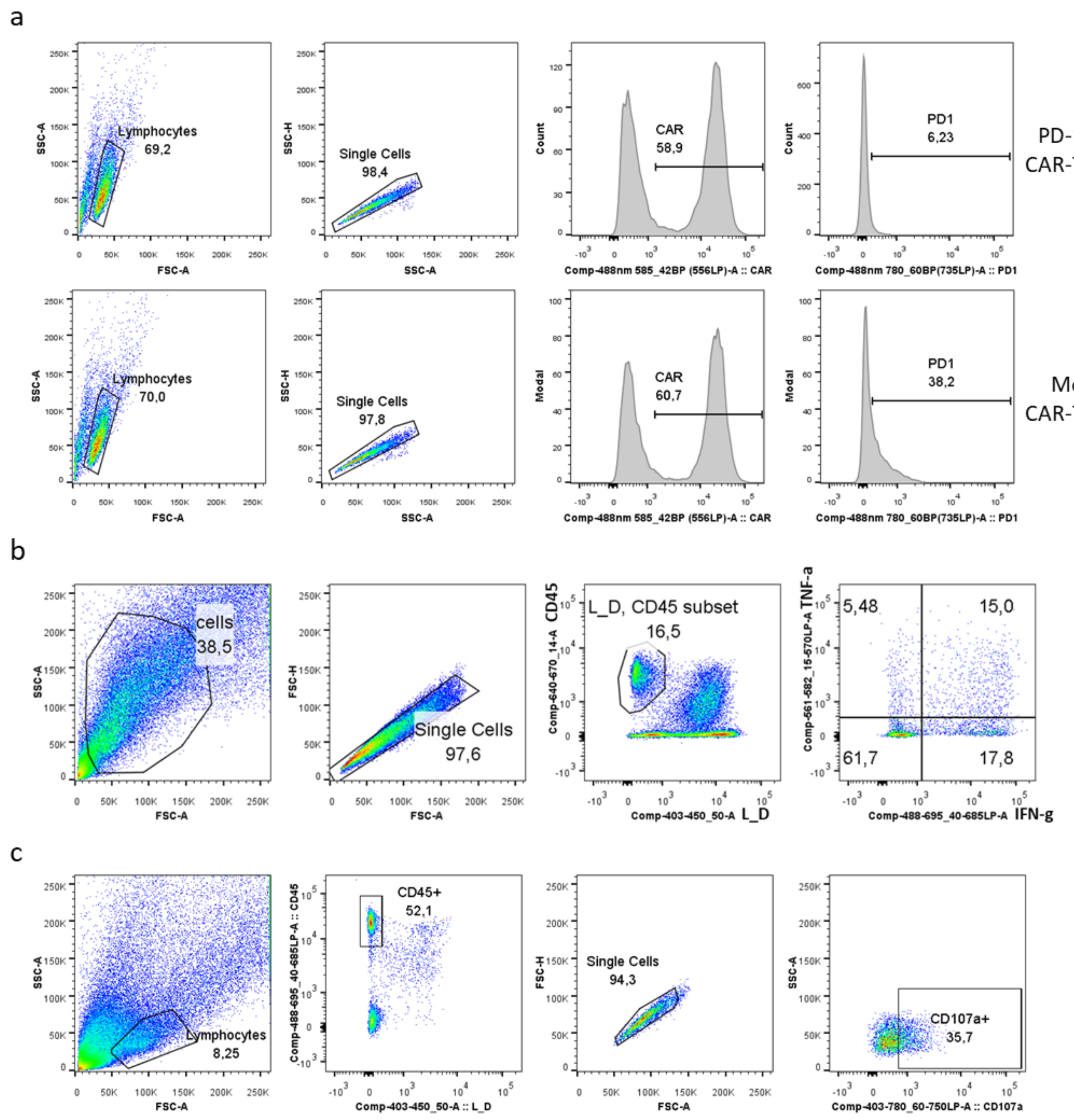

Representative gating strategies shown for **a** CAR and PD-1 staining (as in Fig. 1d-e), **b** intracellular cytokine staining assays (as in Fig. 5g-h, Fig. 7e-f), and **c** CD107-α degranulation studies (as in Fig. 6a).

Supplementary Table 1 Detail of cell lines used:

Cell line	Cell type	Catalog #	Obtained from	Media
293FT	human embryonic kidney cells	R70007	ThermoFisher	DMEM (Gibco) supplemented with 10% fetal bovine serum (Sigma, Lot#F4531), Penicillin-Streptomycin (#15070063, ThermoFisher), 10 mM GlutaMax (#35050061, ThermoFisher) and 1% non-essential amino acids (NEAA, #11140050, ThermoFisher)
SKOV3	ovarian cystadenocarcinoma	HTB-77	ATCC	DMEM (Gibco) supplemented with 10% fetal bovine serum (F9965, Lot#1948396, Gibco) and Penicillin-Streptomycin (#15070063, ThermoFisher)
Capan-2	pancreatic adenocarcinoma	HTB-80	ATCC	DMEM/F12 (Gibco) supplemented with 10% fetal bovine serum (F9965, Lot#1948396, Gibco) and Penicillin-Streptomycin (#15070063, ThermoFisher)
MDA-MB-468	triple negative breast cancer	HTB-132	ATCC	DMEM/F12 (Gibco) supplemented with 10% fetal bovine serum (F9965, Lot#1948396, Gibco) and Penicillin-Streptomycin (#15070063, ThermoFisher)
HCC1954	breast ductal carcinoma	CRL-2338	ATCC	RPMI-1640 (Gibco) supplemented with 10% fetal bovine serum (F9965, Lot#1948396, Gibco) and Penicillin-Streptomycin (#15070063, ThermoFisher)
Jurkat	acute T cell leukemia	TIB-152	ATCC	RPMI-1640 (Gibco) supplemented with 10% fetal bovine serum (F9965, Lot#1948396, Gibco) and Penicillin-Streptomycin (#15070063, ThermoFisher)
HPASMC	Human Pulmonary Artery Smooth Muscle Cells	C-12521	Promocell	Smooth Muscle Cell Growth Medium 2 (Promocell)

HPAEC	Human Pulmonary Artery<br>Endothelial Cells	C-12241	Promocell	Endothelial Cell Growth Medium (Promocell)
HREpC	Human Renal Epithelial Cells	C-12665	Promocell	Renal Epithelial Cell Growth Medium 2 (Promocell)
NHEK, single donor	Normal Human Epidermal Keratinocytes	C-12003	Promocell	Keratinocyte Growth Medium 2 (Promocell)

Supplementary Table 2 List of antibodies used:

Target	Fluorochrome	Clone	Dilution	Catalog #	Company
CD45	PerCP-Cy5.5	HI30	1:100	564105	BD Biosciences
CD45	APC	2D1	1:100	17-9459-42	Thermofisher
CD25	PECy7	4E3	1:50	25-0257-42	Thermofiher
CD4	AF488	RPA-T4	1:50	557695	BD Biosciences
CD4	PE	OKT-4	1:100	12-0048-42	ThermoFisher
CD8	APC	HIT8a	1:100	300912	BioLegend
CD8	APC-H7	SK1	1:100	566856	BD Biosciences
PD-1	PECy7	EH12.1	1:50	561272	BD Biosciences
PD-1	APC	J105	1:100	17-2799-42	Thermofisher
PD-1	BV711	EH12.2H7	1:50	329928	BioLegend
PD-L1	APC	29E.2A3	1:100	329708	BioLegend
PD-L1	PE	29E.2A3	1:50	329706	BioLegend
PD-L2	APC	24F.10C12	1:100	329608	BioLegend
TNF-α	PE	MAb11	1:50	554513	BD Biosciences
IFN-γ	PerCP-Cy5.5	B27	1:50	560704	BD Biosciences
CD107a	BV785	H4A3	1:50	328643	Biolegend
HER2	PE	24D2	1:100	324406	BioLegend
FRβ	PE	94b/FOLR2	1:5	391703	BioLegend
anti-mouse IgG, $F(ab')_2$ fragment specific	Biotin-SP (long spacer)	Polyclonal	1:50	115-065-072	Jackson ImmunoResearch
anti-human IgG, $F(ab')_2$ fragment specific	Biotin-SP (long spacer)	Polyclonal	1:50	109-066-006	Jackson ImmunoResearch
Isotype control Mouse IgG1 k	PE	MOPC-21	1:50	555749	BD Biosciences
Isotype control Mouse IgG1 k	APC	MOPC-21	1:100	555751	BD Biosciences
**Related flow cytometry reagents:**					
L/D Fixable viability dye	eFluor450		1:5000	65-0863-14	eBioscience
streptavidin (SA)	PE		1:100	12-4317-87	ThermoFisher
streptavidin (SA)	eFluor450		1:100	48-4317-82	ThermoFisher
