# Supplementary material for: CAR affinity modulates the sensitivity of CAR-T cells to PD-1/PD-L1-mediated inhibition
Source: Nat Commun. 2024 Apr 26;15:3552. doi: 10.1038/s41467-024-47799-z (PMC11053011; doi:10.1038/s41467-024-47799-z)
Supplement: Supplementary file 3 — Reporting Summary [file 41467_2024_47799_MOESM3_ESM.pdf]

nature portfolio

Corresponding author(s): Alba Rodriguez-Garcia and Sonia Guedan

Last updated by author(s): Apr 10, 2024

Reporting Summary

Nature Portfolio wishes to improve the reproducibility of the work that we publish. This form provides structure for consistency and transparency in reporting. For further information on Nature Portfolio policies, see our Editorial Policies and the Editorial Policy Checklist.

Statistics

For all statistical analyses, confirm that the following items are present in the figure legend, table legend, main text, or Methods section.

n/a	Confirmed	
☐	☒	The exact sample size (*n*) for each experimental group/condition, given as a discrete number and unit of measurement
☐	☒	A statement on whether measurements were taken from distinct samples or whether the same sample was measured repeatedly
☐	☒	The statistical test(s) used AND whether they are one- or two-sided<br>*Only common tests should be described solely by name; describe more complex techniques in the Methods section.*
☒	☐	A description of all covariates tested
☐	☒	A description of any assumptions or corrections, such as tests of normality and adjustment for multiple comparisons
☐	☒	A full description of the statistical parameters including central tendency (e.g. means) or other basic estimates (e.g. regression coefficient) AND variation (e.g. standard deviation) or associated estimates of uncertainty (e.g. confidence intervals)
☐	☒	For null hypothesis testing, the test statistic (e.g. *F*, *t*, *r*) with confidence intervals, effect sizes, degrees of freedom and *P* value noted<br>*Give P values as exact values whenever suitable.*
☒	☐	For Bayesian analysis, information on the choice of priors and Markov chain Monte Carlo settings
☒	☐	For hierarchical and complex designs, identification of the appropriate level for tests and full reporting of outcomes
☒	☐	Estimates of effect sizes (e.g. Cohen's *d*, Pearson's *r*), indicating how they were calculated

Our web collection on statistics for biologists contains articles on many of the points above.

Software and code

Policy information about availability of computer code

Data collection	Flow cytometry was collected using BD FACSDiva software (BD). Absorbance in ELISA assays were measured using Gen5 2.07 (Biotek) or iControl 2.0 (LifeSciences) software. Transcriptomic data was collected by nCounter SPRINT profiler v2.3.0.44 (Nanostring).
Data analysis	All statistical calculations were performed using GraphPad Prism 9.4.1 (GraphPad Software Inc.). ICE v3.0 software (Synthego) was used for analysis of KO efficiencies. Flow cytometry results were analyzed by FlowJo Software v10 (TreeStar Inc.). IsoSpeak 2.9.0 software was used for single cell secretome analysis. Rosalind software was used for analysis of nanostring gene expression assays (www.rosalind.bio/nanostring). Enrichr online software (https://maayanlab.cloud/Enrichr/) was used for pathway analysis of transcriptomic data.

For manuscripts utilizing custom algorithms or software that are central to the research but not yet described in published literature, software must be made available to editors and reviewers. We strongly encourage code deposition in a community repository (e.g. GitHub). See the Nature Portfolio guidelines for submitting code & software for further information.

Data

Policy information about availability of data

All manuscripts must include a data availability statement. This statement should provide the following information, where applicable:

- Accession codes, unique identifiers, or web links for publicly available datasets
- A description of any restrictions on data availability
- For clinical datasets or third party data, please ensure that the statement adheres to our policy

The authors declare that all data supporting the results in this study are available within the paper and its Supplementary information. Transcriptomics data have

been deposited in the Gene Expression Omnibus database under accession number GSE252036. Other data are available from the corresponding authors upon reasonable request. Source data are provided with this paper.

Research involving human participants, their data, or biological material

Policy information about studies with human participants or human data. See also policy information about sex, gender (identity/presentation), and sexual orientation and race, ethnicity and racism.

Reporting on sex and gender	N/A
Reporting on race, ethnicity, or other socially relevant groupings	N/A
Population characteristics	Healthy donors
Recruitment	Volunteers from the Barcelona Public Blood and Tissue Bank
Ethics oversight	Human T cells are isolated from buffy coats obtained from the Barcelona Public Blood and Tissue Bank. All samples are deidentified prior to receipt and no protected health information is transferred from the blood bank to our team or institution. Therefore, informed consent is not required from our side. Specific approval for this project was obtained from the local ethic committee (Comité de Ética de la investigación con medicamentos CEIm).

Note that full information on the approval of the study protocol must also be provided in the manuscript.

Field-specific reporting

Please select the one below that is the best fit for your research. If you are not sure, read the appropriate sections before making your selection.

☒ Life sciences ☐ Behavioural & social sciences ☐ Ecological, evolutionary & environmental sciences

For a reference copy of the document with all sections, see nature.com/documents/nr-reporting-summary-flat.pdf

Life sciences study design

All studies must disclose on these points even when the disclosure is negative.

Sample size	No statistical methods were used to pre-determine sample sizes, but all major in vitro experiments were performed with at least three biologcal replicates (i.e. CAR-T cells generated from three different donors) and technical duplicates, to ensure findings were reproducible. Based on prior experience, we estimated that statistically meaningful differences between groups in in vivo experiments would require a sample size of at least 8 tumors (4 mice) per treatment group.
Data exclusions	For in vivo studies, mice with extreme tumor burdens (either too high or too low compared to the average) were excluded from the experiment before CAR T cell transfer. Mice that received unsuccessful i.v. CAR T cell administrations were also excluded.
Replication	Most experiments were performed with at least three biological replicates and technical duplicates, unless otherwise specified. Attempts at experimental replication were successful and support conclusions stated in the manuscript.
Randomization	Mice were randomized prior to CAR-T treatment to ensure equivalent tumor burden among groups. Randomization for in vitro experiments is not applicable.
Blinding	Staff that conducted animal studies were blinded to the group allocation during treatment administration, tumor measurement, and data analysis (tumor volume calculation). Blinding does not apply to in vitro studies.

Reporting for specific materials, systems and methods

We require information from authors about some types of materials, experimental systems and methods used in many studies. Here, indicate whether each material, system or method listed is relevant to your study. If you are not sure if a list item applies to your research, read the appropriate section before selecting a response.

Materials & experimental systems

n/a	Involved in the study	
	☒	Antibodies
	☒	Eukaryotic cell lines
☒		Palaeontology and archaeology
	☒	Animals and other organisms
☒		Clinical data
☒		Dual use research of concern
☒		Plants

Methods

n/a	Involved in the study	
☒		ChIP-seq
	☒	Flow cytometry
☒		MRI-based neuroimaging

Antibodies

Antibodies used

CD45 PerCP-Cy5.5 HI30 1:100 564105 BD Biosciences
CD45 APC 2D1 1:100 17-9459-42 Thermofisher
CD25 PECy7 4E3 1:50 25-0257-42 Thermofiher
CD4 AF488 RPA-T4 1:50 557695 BD Biosciences
CD4 PE OKT-4 1:100 12-0048-42 ThermoFisher
CD8 APC HIT8a 1:100 300912 BioLegend
CD8 APC-H7 SK1 1:100 566856 BD Biosciences
PD-1 PECy7 EH12.1 1:50 561272 BD Biosciences
PD-1 APC J105 1:100 17-2799-42 Thermofisher
PD-1 BV711 EH12.2H7 1:50 329928 BioLegend
PD-L1 APC 29E.2A3 1:100 329708 BioLegend
PD-L1 PE 29E.2A3 1:50 329706 BioLegend
PD-L2 APC 24F.10C12 1:100 329608 BioLegend
TNF-α PE MAb11 1:50 554513 BD Biosciences
IFN-γ PerCP-Cy5.5 B27 1:50 560704 BD Biosciences
CD107a BV785 H4A3 1:50 328643 Biolegend
HER2 PE 24D2 1:100 324406 BioLegend
FRβ PE 94b/FOLR2 1:5 391703 BioLegend
anti-mouse IgG, $F(ab')_2$ fragment specific Biotin-SP (long spacer) Polyclonal 1:50 115-065-072 Jackson ImmunoResearch
anti-human IgG, $F(ab')_2$ fragment specific Biotin-SP (long spacer) Polyclonal 1:50 109-066-006 Jackson ImmunoResearch
Isotype control Mouse IgG1 k PE MOPC-21 1:50 555749 BD Biosciences
Isotype control Mouse IgG1 k APC MOPC-21 1:100 555751 BD Biosciences
Related flow cytometry reagents:
L/D Fixable viability dye eFluor450 1:5000 65-0863-14 eBioscience
streptavidin (SA) PE 1:100 12-4317-87 ThermoFisher
streptavidin (SA) eFluor450 1:100 48-4317-82 ThermoFisher

For immunohistochemistry the following antibody was used: rabbit anti-human Anti-PD-L1 antibody (15165T, Cell signaling, dil 1:100)

Validation

All antibodies used in this study were commercial and validated by the manufacturer for use to detect human species targets. Species, application validations and citations for primary antibodies can be found from the manufacturer's websites.
https://www.bdbiosciences.com/en-eu/products/reagents/flow-cytometry-reagents/research-reagents/single-color-antibodies-ruo/percp-cy-5-5-mouse-anti-human-cd45.564105
https://www.thermofisher.com/antibody/product/CD45-Antibody-clone-2D1-Monoclonal/17-9459-42
https://www.thermofisher.com/antibody/product/CD25-Antibody-clone-CD25-4E3-Monoclonal/25-0257-42
https://www.bdbiosciences.com/en-es/products/reagents/flow-cytometry-reagents/research-reagents/single-color-antibodies-ruo/alexa-fluor-488-mouse-anti-human-cd4.557695
https://www.thermofisher.com/antibody/product/CD4-Antibody-clone-OKT4-OKT-4-Monoclonal/12-0048-42
https://www.biolegend.com/fr-ch/products/apc-anti-human-cd8a-antibody-759
https://www.bdbiosciences.com/en-us/products/reagents/flow-cytometry-reagents/research-reagents/single-color-antibodies-ruo/apc-h7-mouse-anti-human-cd8.560179
https://www.bdbiosciences.com/en-es/products/reagents/flow-cytometry-reagents/research-reagents/single-color-antibodies-ruo/pe-cy-7-mouse-anti-human-cd279-pd-1.561272
https://www.thermofisher.com/antibody/product/CD279-PD-1-Antibody-clone-eBioJ105-J105-Monoclonal/17-2799-42
https://www.biolegend.com/de-at/products/brilliant-violet-711-anti-human-cd279-pd-1-antibody-7945
https://www.biolegend.com/fr-ch/products/apc-anti-human-cd274-b7-h1-pd-l1-antibody-4376?GroupID=BLG5404
https://www.biolegend.com/en-gb/products/pe-anti-human-cd274-b7-h1-pd-l1-antibody-4375?GroupID=BLG5402
https://www.biolegend.com/nl-nl/products/apc-anti-human-cd273-b7-dc-pd-l2-antibody-4694?GroupID=BLG5710
https://www.bdbiosciences.com/en-eu/products/reagents/flow-cytometry-reagents/research-reagents/single-color-antibodies-ruo/pe-mouse-anti-human-tnf.554513
https://www.bdbiosciences.com/en-es/products/reagents/flow-cytometry-reagents/research-reagents/single-color-antibodies-ruo/

percp-cy-5-5-mouse-anti-human-ifn.560704
https://www.biolegend.com/ja-jp/products/brilliant-violet-785-anti-human-cd107a-lamp-1-antibody-12095
https://www.biolegend.com/en-ie/products/pe-anti-human-cd340-erbb2-her-2-antibody-3766?GroupID=BLG5136
https://www.biolegend.com/nl-be/products/pe-anti-human-folate-receptor-beta-fr-beta-antibody-15115?GroupID=GROUP389
https://www.jacksonimmuno.com/catalog/products/115-065-072
https://www.jacksonimmuno.com/catalog/products/109-066-006
https://www.bdbiosciences.com/en-es/products/reagents/flow-cytometry-reagents/research-reagents/flow-cytometry-controls-and-lysates/pe-mouse-igg1-isotype-control.555749
https://www.bdbiosciences.com/en-us/products/reagents/flow-cytometry-reagents/research-reagents/flow-cytometry-controls-and-lysates/apc-mouse-igg1-isotype-control.555751

Eukaryotic cell lines

Policy information about cell lines and Sex and Gender in Research

Cell line source(s)	All human cancer cell lines were purchased from the American Type Culture Collection (ATCC) except for HEK 293FT (human embryonic kidney cells), which was obtained from Thermo Fisher Scientific (ThermoFisher). All human healthy primary cells were purchased from Promocell. SKOV3 PD-L1 KO, SKOV3 PD-L1 Low, SKOV3 PD-L1 high, SKOV3.FRb PD-L1 KO, SKOV3.FRb PD-L1 high, MDA-MB-468 HER2 low, MDA-MB-468 HER2 High, MDA-MB-468 PD-L1 High HER2 Low and MDA-MB-468 PD-L1 High HER2 High were engineered in the lab.
Authentication	All cancer cell lines were authenticated in 2019 by IDEXX Bioanalytics using the Human 9-Marker STR Profile.
Mycoplasma contamination	All cell lines were routinely tested for mycoplasma contamination. No mycoplasma contamination was found.
Commonly misidentified lines (See ICLAC register)	None of the cell lines used in this study are listed in the ICLAC Database of Cross-contaminated or Misidentified Cell Lines.

Animals and other research organisms

Policy information about studies involving animals; ARRIVE guidelines recommended for reporting animal research, and Sex and Gender in Research

Laboratory animals	NOD/SCID/IL2-receptor γ chain knockout (NSG) mice were purchased from Jackson Laboratory. Mice were bred and maintained within the Animal Facility at the University of Barcelona, where their health status was regularly monitored by qualified personnel. 6-8 week old mice were used for tumor implantation.
Wild animals	The study did not involve wild animals.
Reporting on sex	Female mice were used for ovarian cancer (SKOV3) and breast cancer (HCC1954) models. Male mice were used for the pancreatic cancer model (CAPAN2).
Field-collected samples	The study did not involve samples collected in the field.
Ethics oversight	All animal studies were approved by the Ethic Committee for Animal Experimentation (CEEA) of the University of Barcelona and Generalitat de Catalunya. All studies were conducted under the approved protocol IDIBAPS (184/20) under established policies at the University of Barcelona.

Note that full information on the approval of the study protocol must also be provided in the manuscript.

Flow Cytometry

Plots

Confirm that:

- [x] The axis labels state the marker and fluorochrome used (e.g. CD4-FITC).
- [ ] The axis scales are clearly visible. Include numbers along axes only for bottom left plot of group (a 'group' is an analysis of identical markers).
- [x] All plots are contour plots with outliers or pseudocolor plots.
- [x] A numerical value for number of cells or percentage (with statistics) is provided.

Methodology

Sample preparation	For tumor cell lines, cells were first detached with Tryple Select (Gibco) or Versene (Gibco) when required before staining. For T cells, single-cell suspensions were stained with antibodies according to the manufacturer's protocols.
Instrument	Flow cytometry was performed on a BD FACS CANTO 3L, BD LSRFortessa 5L, and BD LSRFortessa 4L. Sorting was performed on a BD FACSAria II and a BD FACSAria SORP (Becton-Dickinson).

Software	FCS files were analyzed with FlowJo v10.
Cell population abundance	Target cell lines and CAR-T cells were sorted for >99% purity before extracting RNA on a BD FACSAria II or a BD FACSAria SORP by the staff of the Cytometry and Cell Sorting Facility (IDIBAPS). For gating strategies, at least 10000 cells were analyzed.
Gating strategy	Tipically, cells were first gated by FSC and SSC plots, then single cells were selected by FSC-H vs FSC-A. Finally, prior to the gate of interest, live cells were selected as defined by Live/Dead stain negativity. Detailed gating strategies were provided in figures. Positive and negative gates were determined based on isotype or untransduced controls. For gating on CAR expression, untransduced T cells were used to draw the gates of interest.

[x] Tick this box to confirm that a figure exemplifying the gating strategy is provided in the Supplementary Information.
